# Cdk4 Regulates Glioblastoma Cell Invasion and Stemness and Is Target of a Notch Inhibitor Plus Resveratrol Combined Treatment

**DOI:** 10.3390/ijms241210094

**Published:** 2023-06-13

**Authors:** Francesca Giordano, Maria D’Amico, Francesca Ida Montalto, Rocco Malivindi, Adele Chimento, Francesca Luisa Conforti, Vincenzo Pezzi, Maria Luisa Panno, Sebastiano Andò, Francesca De Amicis

**Affiliations:** 1 Department of Pharmacy, Health and Nutritional Sciences, University of Calabria, 87036 Rende, Italymaria.damico@unical.it (M.D.);; 2 Health Center, University of Calabria, 87036 Rende, Italy

**Keywords:** brain tumors, Notch, stemness, EMT, paxillin, cyclin D1

## Abstract

Glioblastoma multiforme (GBM) is one of the most aggressive types of cancer characterized by poor patient outcomes. To date, it is believed that the major cause of its recurrence and chemoresistance is represented by the enrichment of GBM stem cells (GSCs) sustained by the abnormal activation of a number of signaling pathways. In this study, we found that in GBM cells, treatment with low toxicity doses of the γ-secretase inhibitor RO4929097 (GSI), blocking the Notch pathway activity, in combination with resveratrol (RSV) was able to reverse the basal mesenchymal phenotype to an epithelial-like phenotype, affecting invasion and stemness interplay. The mechanism was dependent on cyclin D1 and cyclin-dependent kinase (CDK4), leading to a reduction of paxillin (Pxn) phosphorylation. Consequently, we discovered the reduced interaction of Pxn with vinculin (Vcl), which, during cell migration, transmits the intracellular forces to the extracellular matrix. The exogenous expression of a constitutively active Cdk4 mutant prevented the RSV + GSI inhibitory effects in GBM cell motility/invasion and augmented the expression of stemness-specific markers, as well as the neurosphere sizes/forming abilities in untreated cells. In conclusion, we propose that Cdk4 is an important regulator of GBM stem-like phenotypes and invasive capacity, highlighting how the combined treatment of Notch inhibitors and RSV could be prospectively implemented in the novel therapeutic strategies to target Cdk4 for these aggressive brain tumors.

## 1. Introduction

Glioblastoma multiforme (GBM) is one of the most lethal tumor types, characterized by a very low 5-year survival rate [1]. The unfortunate prognosis of GBM patients and the high rate of recurrence are related to the marked intra-tumoral cellular heterogeneity and plasticity, together with the infiltrative and migratory phenotype of GBM cells, capable of invading diffusely into normal brain parenchyma [2]. During invasion, GBM cells become polarized, and dynamic cytoskeletal rearrangements at their leading edge are required, mainly involving focal adhesion kinases (Fak) and integrins [3]. To form focal adhesion contacts, integrins recruit a diverse array of intracellular proteins, among them the adaptor protein paxillin (Pxn) [4,5] and the cytoplasmic cyclin D1 (Ccnd1), its binding partner the cyclin-dependent kinase 4 (Cdk4) [6], and vinculin (Vcl) [7]. Vcl influences cell morphology but also coordinates the contractile forces resulting from a highly dynamic intracellular protein network, able to integrate multiple signals shaping cell-invasive behavior. It has been documented that cell invasion may be critically dependent on the acquisition of the epithelial mesenchymal transition (EMT)-associated phenotype [8]. Interestingly, cancer cells undergoing a hybrid epithelial/mesenchymal phenotype may acquire stem-like features; thus, EMT has been closely linked to cancer stemness [9]. Cancer stem cells (CSCs) show increased tumor-propagating potential following their transplantation into immunodeficient mice. Interestingly, forced expression of transcription factors that promote EMT, such as TWIST1 or SNAIL1, in mammary epithelial cells increases their ability to give rise to secondary tumors upon resection [10]. Since the tumorigenic and self-renewing GBM stem cells (GSCs) contribute to tumor initiation and therapeutic resistance, a deeper understanding of the underlying molecular processes that drive cancer stemness and invasiveness maintenance will enhance our ability to selectively target and ablate cell populations becoming resistant to different drugs. 

A number of molecular mechanisms have been identified that contribute to the therapeutic resistance of GSCs to current therapies, including DNA damage checkpoint, the Wnt signaling cascade, phosphatidylinositol 3-kinases (PI3K), NF-κB, histone methyltransferase such as EZH2, and, more recently, Notch [11].

The Notch pathway controls migration, self-renewal, and differentiation in different cell types [12]. The activation of Notch signaling confers a growth advantage by keeping tumor cells in a stem cell-like proliferative state [13]. In contrast, pharmacological Notch blockade can induce protective autophagy in glioma neurospheres [14]. The Notch pathway has been targeted in vivo using gamma-secretase inhibitors (GSIs) [15], alone or in combination regimens, which may be useful in counteracting multiple mechanisms of CSCs resistance. Our recent studies demonstrate the synergistic effect of RO4929097 (GSI) in combination with resveratrol (RSV; 3,5,40-trihydroxy-trans-stilbene) to induce the autophagy blockade in GBM cell lines [16]. RSV inhibits GBM cell motility and invasiveness through a huge number of molecular targets and signaling pathways such as MMP-2 and the RhoA/ROCK signaling pathway [17,18]. 

Herein, we investigate a novel mechanism by which RSV + GSI cotreatment may hamper the link existing between EMT and the acquisition of stem-like properties, affecting the invasion and stemness interplay in GBM cells. We demonstrate that Cdk4 plays an essential role in this combined network, proposing that Cdk4 can enable cells in the epithelial/mesenchymal states to gain stemness. These cancer properties were reversed by RSV + GSI combined treatment.

## 2. Results

### 2.1. RSV and GSI in Combination Suppress the Motility and Invasion in GBM Cells

**Figure 1:**
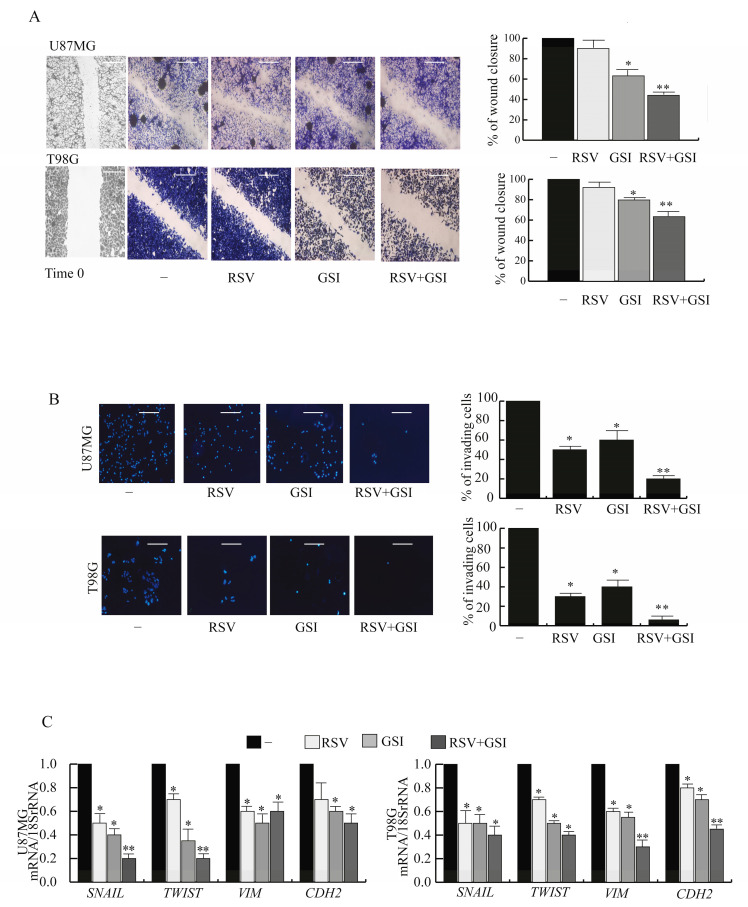
Effects of RSV and GSI cotreatment on U87 MG and T98G cell motility and invasion. (**A**). Left panel. Wound healing assays in U87 MG and T98G cells treated for 24 h with vehicle (-) or 10 μM RSV and/or 2 μM GSI. Images are representative of three independent experiments. Original magnification, ×20. Scale bars = 200 μm Right panel. The histograms represent the relative percentage of wound closure versus (vs.) vehicle, calculated by ImageJ software version 1.51q. Data are expressed as means ± SD of three different experiments. * *p* < 0.05 vs. vehicle (-), ** *p* < 0.05 vs. RSV- or GSI-treated cells. Small squares, time 0. (**B**). Boyden chamber invasion assays. Left panel. U87MG and T98G cells were treated for 24 with vehicle (-) or 10 μM RSV and/or 2 μM GSI and seeded in the chambers. After 8 h, cells were fixed and stained. Images are representative of three independent experiments. Original magnification, ×20. Scale bars = 50 μm. Right panel. The histograms represent the relative percentage of invaded cells. Data are expressed as means ± SD of three different experiments. * *p* < 0.05 vs. vehicle (-), ** *p* < 0.05 vs. RSV- or GSI-treated cells. (**C**). Real-time RT-PCR assay. U87MG and T98G cells were treated for 24 h with vehicle (-) or 10 μM RSV and/or 2 μM GSI. Data are expressed as means ± SD of three different experiments, each performed in triplicate. * *p* < 0.05 vs. vehicle (-); ** *p* < 0.05 vs. RSV- or GSI-treated cells.

To assess the motility and invasion of U87MG and T98G GBM cells, several studies were performed. Results of wound-healing assays showed that U87MG cells moved towards the wound areas more quickly than T98G cells (Figure 1A). The combination of RSV plus GSI compromised the wound closure in both cell types compared to separate treatment with either RSV or GSI alone. Additionally, numerous GBM cells migrated across the Matrigel membrane in transwell assays in the untreated control (Figure 1B). In contrast, significantly fewer GBM cells passed through the membrane after cotreatment, compared to single treatment with either RSV or GSI alone, indicating anti-invasion effects of this regimen. These described events appear to be the biological correlate of the RSV plus GSI inhibitory effects on the expression of genes regulating EMT [19,20], such as the transcriptional repressors SNAIL and TWIST, as well as the mesenchymal markers vimentin (VIM) and N-cadherin (CDH2), compared with the control or RSV or GSI alone (Figure 1C). Thus, the mesenchymal phenotype of the GBM cells was suppressed by RSV + GSI stimulus. 

### 2.2. Pxn Phosphorylation and Cdk4/Ccnd1 Are Affected by RSV and GSI to Inhibit GBM Cell Invasion

**Figure 2:**
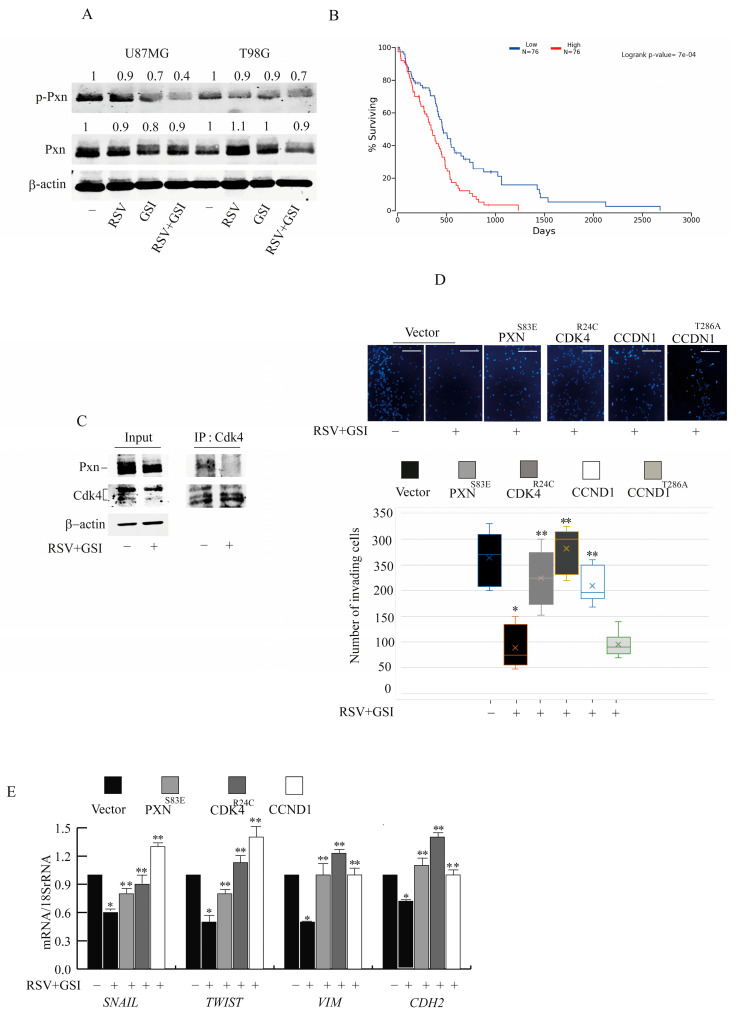
Effects of RSV and GSI cotreatment on Pxn phosphorylation and EMT markers (**A**). U87MG and T98G cells were treated with vehicle (-) or 10 μM RSV and/or 2 μM GSI for 24 h, and immunoblotting was carried out. β-actin was used as the loading control. Images show the results of one representative experiment out of three. Numbers above the blots represent the fold change of quantified value versus control normalized for β-actin. (**B**). Kaplan–Meier overall survival (% surviving) and logrank *p* value. Kaplan–Meier survival analysis in GBM patients (n = 76) with high and low Pxn expression analyzed as described in the Materials and Methods. (**C**). Coimmunoprecipitation analysis. Protein extracts from T98G cells treated with vehicle (-) or RSV + GSI for 24 h were used for input or for immunoprecipitation (IP) by using a specific anti-Cdk4 antibody as indicated. Filters were blotted for the indicated proteins. β-actin was used as the loading control. Images show the results of one representative experiment out of three. (**D**). T98G cells were transfected with vector, PXN^S83E^, CDK4^R24C^ mutant, CCDN1, or CCDN1^T286^ expression vector and treated with vehicle (-) or 10 μM RSV + 2 μM GSI for 24 h, and Boyden chamber invasion assay was performed. Left panel. Images are representative of three independent experiments. Original magnification, ×20. Scale bars = 50 μm. Right panel. Box-and-whisker plots represent values from minimum to maximum. Top and bottom whiskers denote upper (Q3) and lower (Q1) quartiles, respectively. Boxes refer to interquartile ranges. Medians are denoted as the horizontal line in the middle of the boxes. * *p* < 0.05 vs. (-); ** *p* < 0.05 vs. Vector RSV + GSI. (**E**). Real-time RT-PCR assay for SNAIL, TWIST; VIM and CDH2. T98G cells were transfected with vector, PXN^S83E^, CDK4^R24C^ mutant, or CCDN1 expression vector, treated with vehicle (-) or 10 μM RSV + 2 μM GSI for 24 h. Data are expressed as means ± SD of three different experiments, each performed in triplicate. * *p* < 0.05 vs. vehicle (-); ** *p* < 0.05 vs. vector RSV + GSI treated cells.

Next, we observed that the combined treatment with RSV + GSI decreased the phosphorylation of Pxn at Ser178, which orchestrates cell migration and focal adhesion maturation [4] (Figure 2A). According to the TGCA GBM dataset, PXN expression assumes a reliable prognostic relevance [21], since GBM patients with high PXN expression show a lower percentage of surviving than those with low PXN expression (Figure 2B). In cancer cells, Pxn phosphorylation is regulated by the kinase activity of Cdk4 and by the cytoplasmic Ccnd1, which colocalize with FA components, thus regulating cell invasion and metastasis [22,23,24]. Co-IP assay validated the endogenous interaction of Cdk4 with Pxn in T98G cells, and RSV + GSI suppressed this interaction (Figure 2C). We previously reported that Cdk4 and Ccdn1 levels were decreased by the RSV + GSI combination [16]. The ectopic over-expression of the constitutively active CDK4^R24C^ mutant, which abolishes the ability of p16 (INK4a) to inhibit Cdk4 [25], restored the invasive potential (Figure 2D) and greatly counteracted the RSV + GSI effects at mRNA levels of genes regulating EMT (Figure 2E). Similar results were obtained by the ectopic expression of PXNS83E (Figure 2D,E) or CCND1 plasmids (Figure 2D,E). Instead, the exogenous expression CCND1T286A (mutated in the phosphorylation site targeting Ccnd1 for nuclear export) failed to counteract RSV + GSI action (Figure 2D).

### 2.3. RSV and GSI in Combination Reduce the Interaction between Paxillin and Vinculin In Vitro

**Figure 3:**
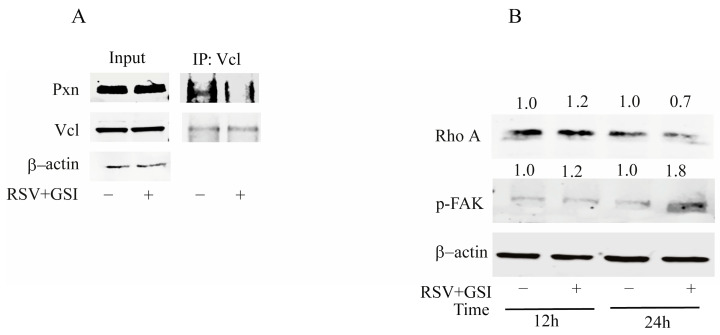
Effects of RSV and GSI cotreatment on Pxn/Vcl interaction. (**A**). Coimmunoprecipitation analysis. Protein extracts from T98G cells treated with vehicle (-) or RSV + GSI for 24 h were used for input or for immunoprecipitation (IP) by using a specific anti-Vcl antibody as indicated. Filters were blotted for the indicated proteins. Β-actin was used as the loading control. Images show the results of one representative experiment out of three. (**B**). T98G cells were treated with vehicle (-) or RSV + GSI as indicated, and immunoblotting was carried out. β-actin was used as the loading control. Images show the results of one representative experiment out of three. Numbers above the blots represent the fold change of quantified value versus control normalized for β-actin.

Phosphorylation of Pxn was implicated in the binding of Vcl, a key component of cytoskeletal scaffold proteins placed in the FA for the transmission of intracellular forces to the extracellular matrix [26], and Pxn/Vcl interaction has been observed in newly established focal complexes during cell migration [27]. Our IP studies evidenced the physical interaction between Pxn and Vcl in T98G cells, which was reduced by the RSV + GSI stimulus (Figure 3A), despite unchanged Vcl expression. The interactions between the FA proteins Vcl and Pxn are spatially regulated by the activity of RhoA and p-Fak, which were modulated after 24 h (h) of cotreatment (Figure 3B). Similar results were obtained in U87MG cells. These data suggest that RSV and GSI combination impairs EMT and invasive capacity in GBM cells targeting Ccnd1/Cdk4/Pxn/Vcl regulators [6,23,24,28].

### 2.4. CCND1 and CDK4 Gene Expression Increases by Switching GBM Cells from Monolayer to Neurospheres Coltures

**Figure 4:**
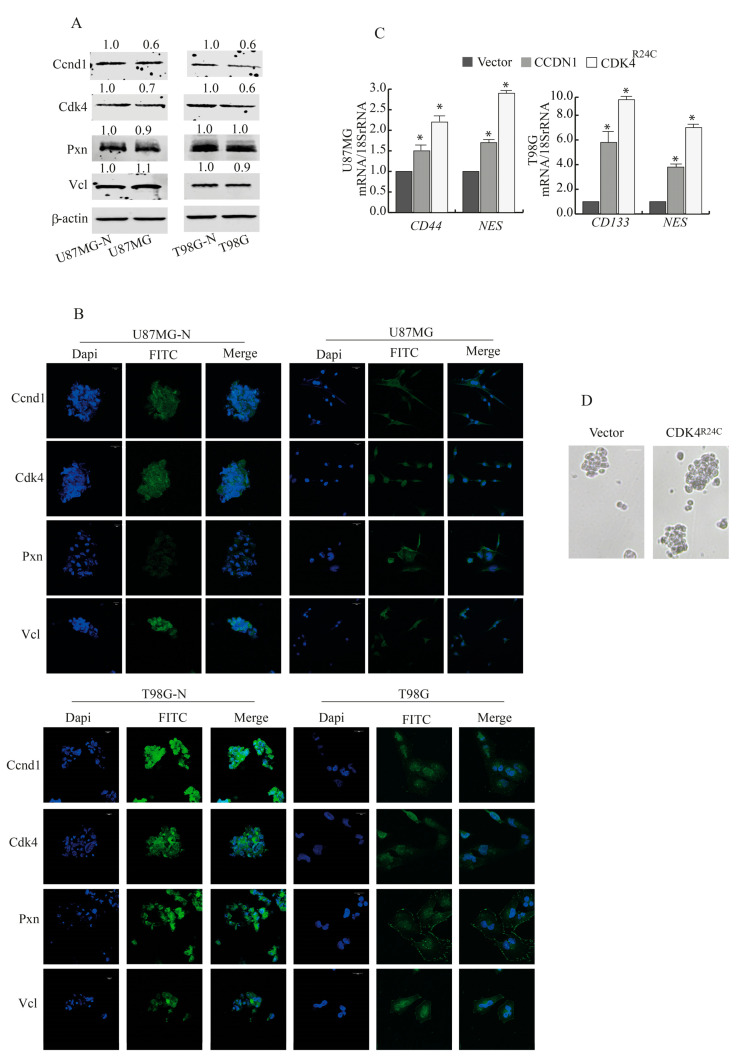
Ccnd1 and Cdk4 expression in GBM cells cultured as neurospheres and as a monolayer (**A**) U87MG and T98G cells were grown as neurospheres (U87MG-N and T98G-N cells, respectively) or as monolayer cultures, and immunoblotting was carried out. β-actin was used as the loading control. Images show the results of one representative experiment out of three. Numbers above the blots represent the fold change of quantified value versus control normalized for β-actin. (**B**). Immunofluorescence images of neurospheres (left panel scale bar = 10 µm) or monolayer (right panel scale bar = 20 µm) stained for Ccdn1, Cdk4, Pxn, and Vcl. DAPI staining for nuclear detection. Original magnification, ×100. (**C**). Real-time RT-PCR assay for CD44, CD133, and NES. U87MG and T98G cells were transfected with vector or CCDN1 or CDK4^R24C^ mutant expression vector. Data are expressed as means ± SD of three different experiments, each performed in triplicate. * *p* < 0.05 vs. vector. (**D**). T98G cells transfected with vector or CDK4^R24C^ mutant expression vector were cultured as neurospheres for 72 h. Images show the results of one representative experiment out of three. Scale bar = 20 µm.

CDK4 regulates cancer cell stemness [29] and has been shown to promote normal stem cell expansion [30]; thus, we examined its participation in the acquisition of stem cell-like properties. To this aim, we determined whether switching U87MG and T98G cells from monolayer to neurosphere (U87MG-N and T98G-N) cultures could have caused the specific mRNA expression of CCND1 and CDK4, together with the expression of specific gene markers of stemness and EMT. Interestingly, CCND1 and CDK4 were significantly upregulated in U87MG-N and T98G-N cultures compared with the monolayer, together with a number of markers classically linked to the acquisition of stem cell characteristics, such as CD133, SOX2, GFAP, and the EMT markers SNAIL and TWIST (Table 1). No substantial change (FC < 1.5 or *p* > 0.05) has been observed for PXN, VCL, VIM, or CDH2 in both cell types, CD44 in T98G, or NANOG in U87MG cells, suggesting the existence of a hybrid phenotype. Immunoblotting analysis (Figure 4A) and immunofluorescence (Figure 4B) confirmed that Ccnd1 and Cdk4 protein expression strongly increased in neurospheres compared with monolayer culture. Pxn and Vcl expression did not substantially change.

We further investigated the role of CCND1 and CDK4 in the appearance of stem-like characteristics. U87MG and T98G cells exogenously overexpressing CCND1 or CDK4R24C mutant showed that while the mRNA of VIM did not change, the levels of additional stem markers such as nestin (NES), CD44, and CD133 respectively increased (Figure 4C). Moreover, exogenous expression of CDK4R24C mutant significantly augmented the neurosphere sizes and forming abilities after 72 h (Figure 4D). These results suggest that the Ccnd1/Cdk4 axis may preserve CD44+ and CD133+ populations and stem-like properties.

### 2.5. RSV and GSI in Combination Affect Stemness of GBM Cells

**Figure 5:**
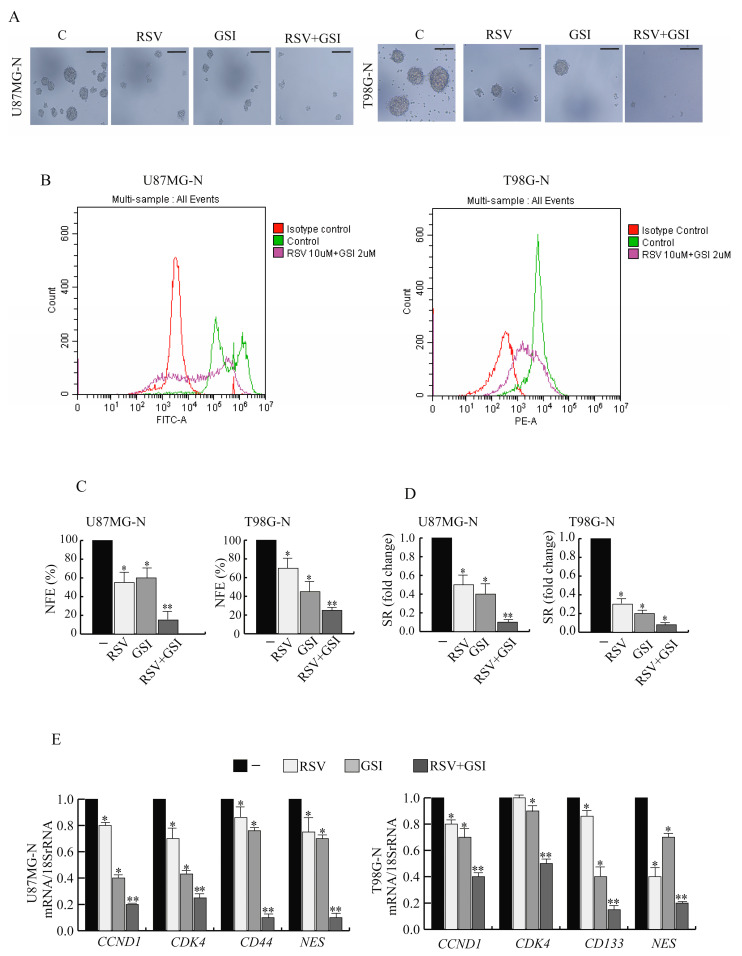
Effects of RSV and GSI cotreatment on glioblastoma stem-like cell phenotype. (**A**) U87MG and T98G cells were grown as neurospheres for 15 days (U87MG-N and T98G-N cells, respectively). Representative phase contrast images of U-87MG-N and T98G-N cells treated with vehicle (-) or 10 μM RSV and/or 2 μM GSI. Scale bar = 50 µm (**B**). Flow cytometry analysis of U87MG-N CD44+ and T98G-N CD133+ subpopulations cultured in the presence of vehicle (-) or RSV + GSI. (**C**). U87MG-N and T98G-N cells cultured in the presence of vehicle (-) or 10 μM RSV and/or 2 μM GSI, and NFE was calculated. Data are expressed as means ± SD of three different experiments, each performed in triplicate. * *p* < 0.05 vs. vehicle (-), ** *p* < 0.05 vs. RSV- or GSI-treated cells. (**D**). U87MG-N and T98G-N cells cultured in the presence of vehicle (-) or 10 μM RSV and/or 2 μM GSI, and SR was calculated. Data are expressed as means ± SD of three different experiments, each performed in triplicate. * *p* < 0.05 vs. vehicle (-), ** *p* < 0.05 vs. RSV- or GSI-treated cells. (**E**). Real-time RT-PCR assay for CCDN1, CDK4, CD44, CD133, and NES. U87MG-N and T98G-N cells were treated with vehicle (-) or 10 μM RSV and/or 2 μM GSI. Data are expressed as means ± SD of three different experiments, each performed in triplicate. * *p* < 0.05 vs. vehicle (-), ** *p* < 0.05 vs. RSV- or GSI-treated cells.

In order to study the possible effect of RSV and GSI, we cultured GBM cells as neurospheres exposed to different stimuli. As shown in (Figure 5A), the relative neurosphere size and neurospheres formation abilities were markedly reduced by RSV + GSI cotreatment compared with vehicle, RSV, or GSI alone. Next, cell populations obtained from U87MG neurosphere cultures were characterized by flow cytometric analysis to investigate the enrichment of neurospheres expressing the CD44 marker (CD44+) after RSV + GSI cotreatment. As shown in Figure 5B, the analysis confirmed that U87MG cells grown as neurospheres contain a high proportion of the CD44+ population, and, interestingly, RSV + GSI treatment led to a significant decrease of the CD44+ subset. Similarly, in T98G-CD133+, cells were reduced following RSV + GSI stimulus. Accordingly, co-treatment significantly inhibited the neurosphere forming efficiency (NFE) (Figure 5C) as well as neurosphere self-renewal (SR) (Figure 5D) compared to the control and cells treated with RSV or GSI alone. Moreover, RSV + GSI-treated neurospheres exhibited decreased mRNA levels of CD44 or CD133, CCND1 or CDK4, and NES, compared with the control and cells treated with RSV or GSI alone (Figure 5E).

## 3. Discussion

Current therapeutic treatments have significantly reduced the mortality in cancer patients. The improvement of patient survival outcome is largely due to the development of targeted therapy for specific tumors [31]. Unfortunately, GBM, the most aggressive form of brain cancer, still displays poor survival outcome, since there is very limited success with the use of conventional chemotherapies along with a lack of targeted therapies [1]. Thus, the identification of novel effectors and signals is still critical for the development of successful treatment for this very aggressive and deadly type of cancer.

The CDK4 gene is frequently amplified in human cancer, and its deregulation has been frequently observed [32]. The founding discoveries in the early 1990s provided proof of principle that Cdk4 inhibition might retard cancer cell development [33]. In this concern, the relationship between CDK4 and clinical prognosis for GBM patients remains unclear. Nevertheless, GSCs behavior is responsible for GBM recurrence, metastasis, and drug resistance, thus playing critical roles in the disease progression [34]. Moreover, several studies suggest the reciprocal regulation of cyclins, Cdks, and Notch to balance stem cell maintenance [35].

In this study, we describe a new function for Cdk4 that is affected by the combination of RSV and the Notch inhibitor (GSI) to inhibit the GSCs population, which display a more invasive phenotype. Specifically, we show that CDK4 and CCDN1 expression is increased by switching towards a stem-like phenotype characterized by the intensification of stem- and EMT-specific markers. The exogenous expression of constitutive active CDK4/CCDN1 is followed by an increase in CD44 and CD133 stem marker expression levels as well as neurosphere size and formation ability. Given that Cdk4 activation regulates cancer stemness and the expression of stem markers, we can speculate that Cdk4 could control the acquisition of hybrid phenotypes, gradually more and more aggressive, characterized by an intermediate molecular state. 

Earlier studies have associated stemness with a high invasive capacity [36]. Particularly, it has been documented that migrating cancer stem cells disseminate by undergoing EMT and retaining stem cell properties, allowing metastatic colony formation [37,38]. Although in GBM, such migrating stem cells are currently under discussion, it was demonstrated that CD133+ GSCs have enhanced invasive capacity when compared to the non-GSC CD133 negative cell fractions [39]. Our results indicate that CDK4 provides a cellular hybrid phenotype endowed with both features, stemness and invasiveness.

The enhanced Pxn phosphorylation by Cdk4 [4] is implicated in the binding of Vcl for the transmission of the intracellular forces [25]. Thus Cdk4, Pxn, and Vcl cooperate to control high motility and invasiveness in GBM cells. 

**Figure 6:**
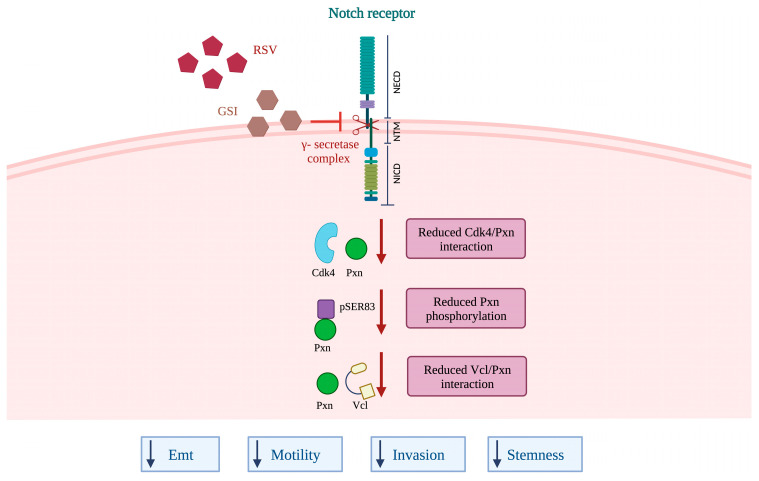
Proposed model for RSV + GSI-induced effects in GBM cells. See text for details.

Therapies targeting developmental pathways such as Notch eliminate neoplastic glioma cells, but their efficacy can be limited by various mechanisms [40]. Up until now, it has emerged that Notch inhibition could be improved when used in conjunction with phytochemical compounds. These combined approaches are frequently more efficacious over traditional single agent therapy regimens for brain tumors since the different drugs affect multiple pathways and cell clones. In such a way, the therapeutic responses may be improved, side effects reduced, and drug resistance prevented [41]. RSV-mediated chemo-sensitization and its multifaceted efficacy in inhibiting the general pro-survival mechanisms are well known [42]. Accordingly, our previous study reports that low toxicity doses of RSV and GSI combination results in the dramatic reduction of Cdk4 levels, together with the induction of GBM cell death and the block of the autophagic flux [16]. Stemming from these findings, the present study validates the rationale of choosing the combination of low toxicity doses of the Notch inhibitor and RSV to inhibit GBM cell migration and invasion. We demonstrate that the mechanism was dependent on the decrease of Pxn phoshorilation, Cdk4-mediated, and Vcl recruitment, thus preventing the tension from being transmitted to nascent adhesion during migration (see Figure 6) [43]. The efficacy of the RSV + GSI combination was determined by the bipartite complex Ccdn1/Cdk4. Interestingly, the restoration of Ccnd1, mutated in the phosphorylation site targeting Ccnd1 for nuclear export, did not rescue the migratory and invasive potential; instead, the exogenous expression of constitutive active Cdk4 (CDK4^R24C^) counteracted RSV + GSI effects. In addition, the single phosphomimetic mutant PXN^S83E^ rescues the invasive potential, confirming that the combined treatment affected migration and invasion through the inhibition of pPXN. 

Recurrent GBM tumors tend to shift to a phenotype with a higher expression of CD44 [44]. Increased CD44 expression correlates with decreased patient survival in 343 patients from Rembrandt data http://rembrandt.nci.nih.gov (accessed on 27 September 2018). CD44+ GSCs possess SR and tumor initiation capacity as well as a drug-resistant nature. Thus, these subpopulations of cancer cells have emerged as potential cellular targets for clinical therapeutic strategies. Furthermore, a recent study in GBM showed that the number of cancer stem cells increased after treatment with the chemotherapeutic drug temozolomide [45]. In the present study, we analyzed the effects of RSV and GSI, alone or in combination, and found that they actually decreased GSCs and stem markers when used in combination [29]. We found that RSV and GSI, reduced SR capacity and GSCs, and exogenous expression of a constitutive active CDK4 abrogated this action. Thus, we reinforced the central role of Cdk4 in GSC generation and as a target of RSV + GSI cotreatment in the reduction of motility and invasion.

In conclusion, in this study, we propose that Cdk4 is an important regulator of GBM stem-like phenotype and invasive capacity, addressing how the Notch inhibitors and RSV combination could therefore be a rational therapeutic strategy to target Cdk4 and to eliminate GSCs, further preventing tumor recurrence in GBM patients.

## 4. Materials and Methods

### 4.1. Chemicals and Reagents

RO4929097 (GSI) (MedChem Express, D.B.A. Italia s.r.l., Segrate, MI, Italy, HY-11102), resveratrol (RSV) (Sigma Aldrich, Merk Life Science S.r.l., Milano, MI, Italy, R5010), aprotinin (SigmaAldrich, Merk Life Science S.r.l. MI, Italy, R5010A-6279), phenylmethylsulfonyl fluoride (PMSF) (Thermo Fischer Scientific, Rodano, MI, Italy, 36978), sodium orthovanadate (Sigma Aldrich, Merk Life Science S.r.l. MI, Italy 450243), NaCl (Sigma Aldrich, Merk Life Science S.r.l. MI, Italy, S9888), MgCl2 (Sigma Aldrich, Merk Life Science S.r.l. MI, Italy M8266), EGTA (Sigma Aldrich, Merk Life Science S.r.l. MI, Italy, 324628), glycerol (Thermo Fischer Scientific, MI, Italy 17904), Triton X-100 (Sigma Aldrich, Merk Life Science S.r.l. MI, Italy, 11332481001), HEPES (Sigma Aldrich, Merk Life Science S.r.l. MI, Italy, H3375). Dulbecco’s Modified Eagle Medium (DMEM-F12) (Thermo Fischer Scientific, MI, Italy, 11039021), Minimum Essential Medium (MEM) (Thermo Fischer Scientific, MI, Italy, 11095080), L-Glutamine (Sigma Aldrich, Merk Life Science S.r.l. MI, Italy, G7513), penicillin/streptomycin (pen/strep) (Sigma Aldrich, Merk Life Science S.r.l. MI, Italy, P0781), Eagle’s nonessential amino acids (VWR, Milano, MI, Italy, IC1681049), sodium pyruvate (Sigma Aldrich, Merk Life Science S.r.l. MI, Italy, S8636), fetal bovine serum (FBS) (Thermo Fischer Scientific, MI, Italy, A5256801), phosphate-buffered saline (PBS) (Thermo Fischer Scientific, MI, Italy, 10010023). Matrigel (Sigma Aldrich, Merk Life Science S.r.l. MI, Italy, 356234). Fibroblast growth factor (FGF) (PeproTech, Thermo Fischer Scientific, MI, Ital, 45033). Antibodies used in this study: anti-p-Pxn (Cell Signaling Technology, D.B.A. Italia s.r.l. MI, Italy, 2541S), anti-Pxn (Cell Signaling Technology, D.B.A. Italia s.r.l. MI, Italy, 2542), anti-Vcl (Cell Signaling Technology, D.B.A. Italia s.r.l. MI, Italy, 4650); anti-Rho a/b/c (Santa Cruz Biotechnology, D.B.A. Italia s.r.l. MI, Italy, sc-418), anti-Ccdn1 (Santa Cruz Biotechnology, D.B.A. Italia s.r.l. MI, Italy, sc-8396), anti-Cdk4 (Santa Cruz Biotechnology, D.B.A. Italia s.r.l. MI, Italy, sc-23896), anti-β-actin (Santa Cruz Biotechnology, D.B.A. Italia s.r.l. MI, Italy, sc-47778), anti- CD133 (Proteintech, D.B.A. Italia s.r.l. MI, Italy, 66666-1-Ig), anti- CD44 (Proteintech, D.B.A. Italia s.r.l. MI, Italy,60224-1-Ig). Protein A/G PLUS agarose (Santa Cruz Biotechnology, D.B.A. Italia s.r.l. MI, Italy, sc-2003).

### 4.2. Cell Culture

Human GBM cell lines U87MG and T98G were purchased from ATCC (Manassas, VA, USA). The U87MG and T98G cells were cultured in MEM, including 10% heat inactivated fetal bovine serum (FBS), 200 mM L-glutamine, 1% penicillin-streptomycin, 1% Eagle’s nonessential amino acids, and 1% sodium pyruvate (Sigma Aldrich, Merk Life Science S.r.l. MI, Italy). Cells were cultured at 37 °C in a humidified atmosphere with 5% carbon dioxide. Cells were stored following the supplier’s recommendations and authenticated every six months after frozen aliquot resuscitations and regularly tested for mycoplasma negativity (MycoAlertMycoplasma Detection Assay, Lonza, Basilea, CH, Switzerland).

### 4.3. Plasmids

pcDNA CCND1 HA, pcDNA CCND1 T286A HA, and CDK4R24C expression vectors were from Addgene (Danvers, MA, 01923 USA). The single phosphomimetic mutant of PXN (PXN S83E) [4] was kindly provided by Dr. E. Gary (Cell Cycle Lab, Institut de Recerca Biomèdica de Lleida and Departament de Ciències Mèdiques Bàsiques; Facultat de Medicina; Universitat de Lleida, 25,198 Lleida, Catalonia, Spain).

### 4.4. Total RNA Extraction, Reverse Transcription PCR, and Real-Time RT-PCR Assay

GBM cells were grown in 100-mm dishes at a density of 2.5 × 10^6^ cells/dish and treated as indicated. Total RNA from cells was extracted using the TRIzol reagent (Thermo Fisher Scientific, MI, Italy, 15596026). Gene expression was assessed by real-time RT-PCR using SYBR Green Universal PCR Master Mix (Bio-Rad, MI, Italy, 1725150), and the relative gene expression levels were assessed and calculated as previously described. [30]. Each sample was normalized on 18S mRNA content. Gene-specific primers sequences: SOX2 forward 5′-CACATGAAGGAGCACCCGGATTAT-3′, reverse 5′ GTTCATGTGCGCGTAACTGTCCAT-3′; NES forward 5′-AACAGCGACGGAGGTCTCTA-3′, reverse 5′-TTCTCTTGTCCCGCAGACTT-3′; GFAP forward 5′-CTGTCCCTAGGTCAGCTTGC, -3′ reverse 5′-GATGTGGAGGGCGATGTAGT-3′; SNAIL forward 5′-CAGACCCACTCAGATGTCAA-3′; SNAIL reverse 5′-CATAGTTAGTCACACCTCGT-3′; TWIST forward 5′-GGGAGTCCGCAGTCTTAC-3′; TWIST reverse 5′-CCTGTCTCGCTTTCTCTTT-3′; CD44 forward 5′ CAGCAACCCTACTGATGATGACG-3′, CD44 reverse 5′-GCCAAGAGGGATGCCAAGATGA-3′; CD133 forward 5′ TTGTGGCAAATCACCAGGTA-3′, CD133 reverse 5′-TCAGATCTGTGAACGCCTTG; CCDN1 forward 5′-CTCCTCGCACTTCTGTTCCTC-3′, CCDN1 reverse 5′-GATGCCAACCTCCTCAACGAC-3′; CDK4 forward 5′-CCATCAGCACAGTTCGTGAGGT-3′, CDK4 reverse 5′-TCAGTTCGGGATGTGGCACAGA; PXN forward 5′-ACAATCGACCCACGTTTCTC-3′, PXN reverse 5′-ATATTGTGCACCGGGATCAT-3′; VCL forward 5′-TGAGCAAGCACAGCGGTGGATT-3′, VCL reverse 5′-TCGGTCACACTTGGCGAGAAGA-3′; CDH2 forward 5′-TCGGTGACAAAGCCCCTGGAT-3′, CDH2 reverse 5′-ACGGCCGTGGCTGTGTTTGAA-3′; VIM forward 5′-GAGAACTTTGCCGTTGAAGC-3′, VIM reverse 5′-GCTTCCTGTAGGTGGCAATC-3′; 18 S forward 5′-GGCGTCCCCCAACTTCTTA-3′ 18 S reverse 5′-GGGCATCACAGACCTGTTATT-3′. The primers were designed using Applied Biosystems Primer Express 2.0 software (Thermo Fisher Scientific, MI, Italy).

#### Protein Isolation

GBM cells were grown in 100-mm dishes at a density of 2.5 × 10^6^ cells/dish and treated as indicated. Cytoplasmic protein lysates were obtained with a buffer containing 50 mM HEPES, pH 7.5, 150 mM NaCl, 1.5 mM MgCl2, 10 mM EGTA, pH 7.5, 10% glycerol, 1% Triton X-100, and protease inhibitors (2 μM sodium orthovanadate, 1% PMSF, 20 μg/mL aprotinin). The expression of different proteins was tested by WB in 50 μg of protein lysates or in 500 μg of immunoprecipitated cell proteins using specific antibodies.

### 4.5. Immunoprecipitation and Immunoblot Analysis

Immunoblot assay [46] and immunoprecipitation [47] were performed as previously described. Odyssey FC (Licor, Lincoln, NE, USA) and Scion Image laser densitometry scanning program Windows version 4.0.3.2 (Scion Corporation, Fredrick, MD, USA) were used, respectively, for acquisition and quantification of each band of interest. Numbers above the blots represent the fold change of quantified value versus control normalized for β-actin.

### 4.6. Transient Transfection Assays

GBM cells were seeded in a 60-mm dish at a density of 7 × 10^4^ cells/dish in regular growth medium and transfected using Lipofectamine reagent (Thermo Fisher Scientific, MI, Italy, 11668030) as recommended by the manufacturer with a mixture containing specific constructs. The Lipofectamine effect was tested in the presence of an empty vector control. After 6 h, the medium was changed for 24 h, and treatments were added as indicated.

### 4.7. Wound-Healing Assays

The method was performed as previously described [48]. Cells were seeded in a 6-well plate at a density of 8 × 10^4^ cells/well. Confluent cell monolayers were scratched and treated as indicated. Wound closure was monitored and quantified. The percentage of wound closure was analyzed by using ImageJ bundled with 64-bit Java 8, quantifying the wound opening area. Among three independent experiments, the most representative one was reported in the picture photographed with an BX51 microscope, 10× objective, OLYMPUS, Milan, MI, Italy.

### 4.8. Invasion Assays

For invasion experiments [49], the inner membrane of Boyden chambers was coated with 30 μL of Matrigel™ Basement Membrane Matrix (BD Biosciences, Becton Dickinson Italia S.p.A. Milan, Italy) and left drying at RT for 30′. The bottom of the well was filled with regular growth media containing 10% FBS. GBM cells seeded in a 60-mm dish at a density of 7 × 10^4^ cells/dish were exposed to various experimental conditions for 24 h. Then, cells were trypsinized and seeded into the top chamber at a density of 15 × 10^4^ cells. The bottom well contained regular growth media containing 10% FBS. After 8 h, U87MG- and T98G-invaded cells were fixed and stained with 4′,6-diamidino-2-phenylindole (DAPI) (Sigma Aldrich, Merk Life Science S.r.l. MI, Italy, D9542). Invasion was quantified in five separate fields/membranes and expressed as the means of invaded cells. Data represent three independent experiments, assayed in triplicate.

### 4.9. Immunofluorescence Assay

For monolayer studies, GBM cells were plated at a density of 12 × 10^4^ cells onto polyL-lysine-coated glass cover slips in MEM with 10% FBS for 18 h. For neurospheres studies, obtained neurospheres sections from frozen O.C.T. compound (Sakura Finetek, 2408 AV Alphen aan den Rijn, The Netherland) blocks using a cryostat were placed into microscope slides. Slides were fixed with 10% neutral buffered formalin (NPF) for 10–15 min, washed three times with 1% serum PBS-T, and incubated with 1% BSA in PBS at room temperature for 1 h. After washing with PBS, sections were incubated with primary antibodies diluted in 1% bovine serum PBS and incubated at room temperature for 2 h and then stored overnight at 4 °C in a humidified chamber. Sections were washed twice with 1% serum PBS-T and incubated with fluorescein isothiocyanate (FITC) conjugated secondary antibodies (Thermofisher Scientific, MI, Italy, 10148543) for 1 h at room temperature. Finally, sections were washed three times with 1% serum PBS-T and incubated with DAPI (1:1000) for 3 min. Images were obtained using an Fluoview FV3000 microscope OLYMPUS MI, Italy, and the images were taken with FV31S-SW software.

### 4.10. Neurosphere Cultures

U87MG and T98G cells were enzymatically and manually disaggregated to obtain a single cell suspension and were plated in ultra-low attachment 100-mm plates (Corning Life Sciences, Corning, NY, USA) at a density of 40 × 10^4^ cells in a serum-free DMEM-F12 supplemented with B27 (Thermo Fischer Scientific, MI, Italy, A3582801), 1 mg/mL penicillin-streptomycin (Life Technologies), 20 ng/mL human epidermal growth factor (EGF, SigmaAldrich, Merk Life Science S.r.l. MI, Italy, E5160), 10 ng/mL basic fibroblast growth factor (FGF, PeproTech, London, UK), and 0.0002% heparin (Sigma Aldrich, Merk Life Science S.r.l. MI, Italy, H0200000). RSV and GSI were added or not at the beginning of the experiments and renewed every 2 days for one week. After 7 days, neurospheres ≥ 50 μm (primary neurospheres) were counted using a microscope (10× magnification), collected, enzymatically dissociated, and plated at the same seeding density as in the primary generation. The neurosphere forming efficiency (NFE) was obtained by dividing the number of neurospheres formed (≥50 μm) by the number of seeded cells and is expressed as the mean percentage of NFE. Self-renewal (SR) was calculated by dividing the total number of secondary neurospheres formed by the total number of primary neurospheres formed and reported as fold change vs. vehicle.

### 4.11. Flow Cytometry

Cells from neurospheres were washed in PBS with 2.5% BSA and labeled with anti-human CD44 or anti-human CD133 and incubated for 2 h at RT followed by incubation with FITC and phycoerythrin (PE-A) conjugated secondary antibody respectively (1 h at RT), according to the supplier’s protocol. A same-isotype antibody was used as a negative control. Cells (10^3^ cells/group) were analyzed using a CytoFLEX (Beckman, Beckman Coulter, Milan, Italy). Data analysis was performed using CytExpert Beckman Coulter software (Beckman Coulter, Milan, Italy).

### 4.12. Statistical Analysis

Each datum point represents the mean ± SD of three different experiments. Data were analyzed for statistical significance (*p* < 0.05) using a two-tailed Student’s *t*-test, performed by the GraphPad-Prism7 software program (GraphPad Inc., San Diego, CA, USA). The Kaplan–Meier survival graph and log-rank *p*-value were calculated using a Kaplan–Meier plotter. (http://www.oncolnc.org/kaplan/?lower=50&upper=50&cancer=GBM&gene_id=5829&raw=pxn&species=mRNA accessed on 20 March 2023) [50].

## Figures and Tables

**Table 1:** Gene expression analysis. Relative expression of CCDN1, CDK4, EMT, and cell stemness-related genes in neurospheres (N) and monolayer cultures estimated by means of RT-qPCR. “FC” is fold change.

Gene Expression Analysis				
Genes	Cell Lines and Cultures			
	U87MG-N/U87MG		T98G-N/T98G	
	FC	*p*-Value	FC	*p*-Value
*CCDN1*	3.6	0.01223	2.1	0.01411
*CDK4*	11.3	0.0111	3.1	0.01155
*PXN*	1.2	>0.99	−1.2	>0.99
*VCL*	−1.1	>0.99	1.1	>0.99
*CD133*	2.7	0.01534	10.2	0.01427
*CD44*	7.7	0.01211	0.1	>0.99
*NES*	2.1	0.01522	4.4	0.01622
*SOX2*	2.8	0.01166	4.7	0.01163
*NANOG*	0.6	>0.99	5.4	0.01554
*GFAP*	4.3	0.01158	3.1	0.01177
*SNAIL*	7.7	0.00166	9.7	0.00143
*TWIST*	3.8	0.00102	5.6	0.01618
*VIM*	1.0	>0.99	1.1	>0.99
*CDH2*	1.3	>0.99	0.4	>0.99

## Data Availability

No new data were created or analyzed in this study. Data sharing is not applicable.
